# Acute Neuromuscular, Physiological and Performance Responses After Strength Training in Runners: A Systematic Review and Meta-Analysis

**DOI:** 10.1186/s40798-022-00497-w

**Published:** 2022-08-17

**Authors:** Gustavo Ivo de Carvalho e Silva, Leandro Henrique Albuquerque Brandão, Devisson dos Santos Silva, Micael Deivison de Jesus Alves, Felipe J. Aidar, Matheus Santos de Sousa Fernandes, Ricardo Aurélio Carvalho Sampaio, Beat Knechtle, Raphael Fabricio de Souza

**Affiliations:** 1 Department of Physical Education, Federal University of Sergipe (UFS), São Cristóvão, Brazil; 2 Graduate Program in Physical Education, Federal University of Sergipe (UFS), São Cristóvão, Sergipe Brazil; 3 Group of Studies and Research of Performance, Sport Health and Paralympic Sports—GEPEPS, Federal University of Sergipe, UFS, São Cristóvão, Sergipe Brazil; 4 Graduate Program in Sports Science, Federal University of Minas Gerais (UFMG), Belo Horizonte, Brazil; 5 Graduate Program in Neuropsychiatry and Behavioral Sciences, Federal University of Pernambuco (UFPE), Recife, Brazil; 6 Institute of Primary Care, University of Zurich, 8091 Zurich, Switzerland; 7 Medbase St. Gallen Am Vadianplatz, 9001 St. Gallen, Switzerland

**Keywords:** Competitive training, Running, Aerobic performance, Strength training

## Abstract

**Background:**

Strength training (ST) is commonly used to improve muscle strength, power, and neuromuscular adaptations and is recommended combined with runner training. It is possible that the acute effects of the strength training session lead to deleterious effects in the subsequent running. The aim of this systematic review and meta-analysis was to verify the acute effects of ST session on the neuromuscular, physiological and performance variables of runners.

**Methods:**

Studies evaluating running performance after resistance exercise in runners in the PubMed and Scopus databases were selected. From 6532 initial references, 19 were selected for qualitative analysis and 13 for meta-analysis. The variables of peak torque (P_*T*_), creatine kinase (CK), delayed-onset muscle soreness (DOMS), rating of perceived exertion (RPE), countermovement jump (CMJ), ventilation (VE), oxygen consumption (VO_2_), lactate (La) and heart rate (HR) were evaluated.

**Results:**

The methodological quality of the included studies was considered reasonable; the meta-analysis indicated that the variables P_*T*_ (*p* = 0.003), DOMS (*p* < 0.0001), CK (*p* < 0.0001), RPE (*p* < 0.0001) had a deleterious effect for the experimental group; for CMJ, VE, VO_2_, La, FC there was no difference. By qualitative synthesis, running performance showed a reduction in speed for the experimental group in two studies and in all that assessed time to exhaustion.

**Conclusion:**

The evidence indicated that acute strength training was associated with a decrease in P_*T*_, increases in DOMS, CK, RPE and had a low impact on the acute responses of CMJ, VE, VO_2_, La, HR and submaximal running sessions.

## Key Points

- Acute strength training (ST) was associated with a decrease in peak torque and an increase in delayed-onset muscle soreness, creatine kinase and rating of perceived exertion in a subsequent running session.
- ST did not affect submaximal running sessions and had little impact on countermovement jump, minute ventilation, oxygen consumption, lactate and heart rate.

## Introduction

To improve physiological, neuromuscular and performance parameters, recreational and professional runner routines include methods of motor skills training including continuous, interval and mixed training [1]. In addition, the specific development of cardiopulmonary capacity is essential to improve gas exchange efficiency, increase maximum oxygen consumption (VO_2_max), lactate threshold, intramuscular glycogen storage capacity and increase mitochondrial density [2], which are conditions important for performance in road running [3].

In addition, the improvement of mechanical (frequency and stride length), neuromuscular (stretch–shortening cycle, muscle–tendon stiffness and muscle strength) and morphological (fiber type distribution) factors improves running economy, differentiating elite runners from long distances [4]. Other mechanisms, such as the ability to generate high power, is important in periods when the athlete performs short sprints during a race to reduce the distance between platoons or for the final sprint [5]. In this sense, strength training (ST) is commonly used to improve neuromuscular adaptation [6, 7] in order to increase anaerobic and speed capacity. Contemporarily, ST is recommended in association with running, parallel to cardiopulmonary training [8].

**Fig. 1:**
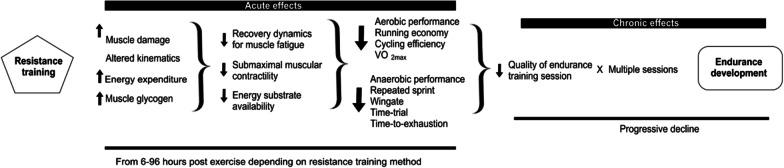
Acute effects of resistance training include increased muscle damage, kinematic alteration, greater energy expenditure, greater neural fatigue, reduced muscle glycogen supply; which lead to worse recovery, less submaximal muscle contractility and less available energy substrate; resulting in a loss of quality of the running session. Finally, this repeated decline in quality can chronically impair the development of endurance capacity (Adapted from Doma et al. [10], with permission

Although there are recommendations for the prescription of ST indicating sessions of two to three times a week, with moderate loads (40–70% RM) without reaching concentric failure [9] or the association of sessions with high loads (> 80%RM) with explosive exercises [5] in programming the combination of different modalities, it is known that a concurrent training session can negatively impact a subsequent session or performance and generate residual acute fatigue [1]. Different physiological processes were raised to explain this process: muscle damage (higher creatine kinase [CK] levels, delayed-onset muscle soreness [DOMS]), kinematic change, higher energy expenditure, neural fatigue and muscle glycogen depletion, which can lead to lower aerobic and anaerobic performance [10] (Fig. 1).

On the other hand, regardless of this understanding, many ST protocols are commonly performed before the practice of running as identified after the warm-up, including cross training exercises [10]**.** The “acute hypothesis” on interference of ST adaptation in endurance development, would better elucidate the impact that individual training sessions have on the level of endurance response during the course of a running competitions or recreational practice in long distance runner [11]. In detail, ST may positively influence parameters that are supposed to be correlated with running performance. Improvements the eccentric–concentric transition including activation an effective stretch–shortening cycle [12].Force development during a short ground contact leading to an increase in stride length [13]. Increase in the recognition of the core musculature, critical for the transfer of energy from the trunk to the smaller extremities, allows a better force transfer to the inferior segments during running [14].

Even though reviews and meta-analyses have already been carried out concerning the chronic adaptations of ST in running economics and runners' performance [5, 8, 9, 15], a systematic review of studies that evaluated the acute response (i.e., immediately after, and at 24 or 48 h a single ST) in indirect and direct variables related to the performance of runners has not been performed and is necessary, since runners and coaches perform acute ST routines without conclusive scientific evidence. We hypothesized that session protocols that require higher training load, higher density and proximity to concentric failure generate greater deleterious effects on the subsequent running session. Therefore, the aim of this systematic review and meta-analysis was to verify the acute effects of a ST session on the neuromuscular, physiological and performance variables of runners.

## Methods

### Search Strategy

A systematic search was carried out with original articles published from 1995 to April 2021, using the PubMed and Scopus databases. Articles written in English language were included and all search results were uploaded to Rayyan's online systematic review management platform. To systematize the search, the Boolean operators (AND and OR) were combined with the following terms: “strength”, “resistance”, “weight training”, “power”, “plyometric”, “concurrent”, “combined strength and endurance”, “exercise”, “training”, “running”, “runner”, “performance”, “time”, “exhaustion”, “speed”, “efficiency”, “endurance”. The systematic review report was carried out based on the “Preferred Reporting Items for Systematic Reviews and Meta-Analyses statement” (PRISMA) [16].

### Inclusion and Exclusion Criteria

The following inclusion criteria were adopted: (1) whether the intervention contained a physical exercise protocol characteristic of ST for the lower limbs; (2) if there was performance evaluation of running in runners (recreational and trained); (3) if the tests used were longer than 75 s; and (4) whether the studies presented the number of participants and all the data needed to calculate the effect size.

**Fig. 2:**
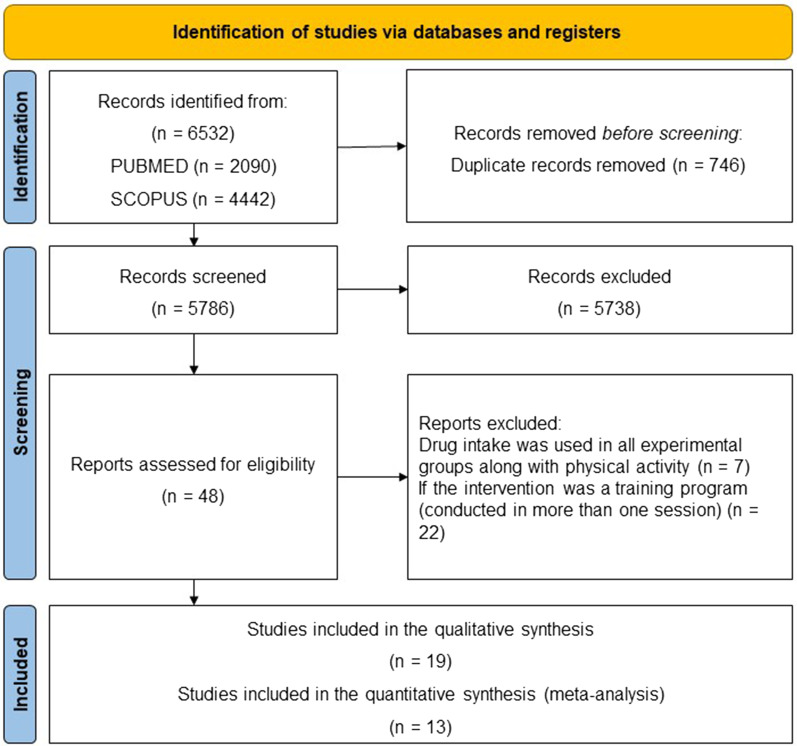
Article search and selection strategy

Articles were excluded when: (1) the full text were not available; (2) texts were not written in English; (3) studies were not performed with humans; (4) drug intake was used in all experimental groups along with physical activity; and (5) if the intervention was a training program (conducted in more than one session). Figure 2 presents the PRISMA flowchart of search and inclusion strategy.

### Eligibility Criteria

Articles that compared the running performance of the experimental groups, which performed a ST session, to that of the control group or to a time prior to the intervention, were eligible for inclusion in the analysis. Studies performed with humans of male sex were selected, regardless of the resistance exercise protocol (traditional, explosive, concurrent, multicomponent, plyometrics, calisthenics). The protocols were chosen for exercise, with training duration above 15 min regardless of exercise intensity: light, moderate or intense loads, submaximal or maximum.

The primary outcome was assessed by evaluating performance on a treadmill or track running tests, running economy, or time to exhaustion on incremental tests. All titles and abstracts were independently reviewed by two investigators to determine study eligibility for inclusion in the review. In case of conflict, a third evaluator was invited. After excluding duplicates, the studies were selected by title and abstract, observing the type of study, the type of population and the type of protocol.

### Quality Assessment

The “Joanna Briggs Institute (JBI) *Critical Appraisal Checklist for Analytical Randomized Controlled Trial and Non-Randomized Experimental Studies*” [17] was used to verify the methodological quality of the articles included. The JBI consists of 8 questions that assess the methodological quality of the articles based on the following criteria: selection of participants, confounding variables, validity and reliability of the results. The questions were answered with “Yes”, “No” or “Undefined”. When the answer was “yes”, a score was given, when the answer was “no” or “undefined”, no score was given. The score for each article was calculated as a percentage and the quality of each study was rated as high (80–100%), fair (50–79%), or low (50%). All studies were independently reviewed by two reviewers. Discrepancies between raters were resolved by consensus.

### Statistical Analysis

The Review Manager statistical program (version 5.3) was used to analyze the primary and secondary outcome data. The results were presented in the form of standardized mean difference (SMD) with 95% confidence intervals (CI) presented by the forest plot. For the analyses, two groups were used: one control group (did not perform a resistance exercise protocol) and one experimental group (which performed resistance exercises). For studies with more than one intervention group, we considered for each comparison only the control versus physical exercise groups. We assessed heterogeneity with the Cochran Test (Ch^2^) and tau-square (tau^2^), measuring the inconsistency (the percentage of the total variation of studies by heterogeneity) of effects during exercise using the *I*^2^ statistic [18]. The level of significance was set at *p* ≤ 0.05 for all analyses.

## Results

### Study Selection

The present review initially identified 6532 articles from the search strategy. From these, 746 were duplicates. Of 5786 articles screened for eligibility, 5,738 were excluded based on title or abstract for reporting an inadequate intervention, had a population that was not runners, and were other types of publication. The full texts of 48 potentially eligible studies were evaluated. Of these, 19 met the criteria and were included in the review, among which 13 made up the meta-analyses (Fig. 2).

### Methodological Quality Assessment

The methodological quality of the included studies was considered reasonable. Most studies presented the inclusion criteria, such as sex, age and questionnaire filling, and all presented the context of the studies. The report was reliably evaluated with valid instruments and trained evaluators; furthermore the objectives are in accordance with the methodological framework. Most studies did not present if they used strategies to identify and eliminate confounding variables (questions 5 and 6). All 19 articles were rated as having a reasonable quality score (50–79%). Table 1 summarizes the quality of the studies.

**Table 1:** Study quality assessment—Joanna Briggs Institute

Studies	Q1	Q2	Q3	Q4	Q5	Q6	Q7	Q8	%
Blagrove et al. 2019 [20]	U	Y	Y	Y	N	N	Y	Y	62.5
Burt et al. 2012 [15]	Y	Y	Y	Y	N	N	Y	Y	75
Burt et al. 2013 [16]	Y	Y	Y	Y	N	N	Y	Y	75
Burt et al. 2014 [17]	Y	Y	Y	Y	N	N	Y	Y	75
De Sousa et al. 2011 [32]	Y	Y	Y	Y	N	N	Y	Y	75
Doma et al. 2013 [25]	Y	Y	Y	Y	N	N	Y	Y	75
Doma et al. 2013 [26]	Y	Y	Y	Y	N	N	Y	Y	75
Doma et al. 2014 [27]	Y	Y	Y	Y	N	N	Y	Y	75
Doma et al. 2015 [28]	Y	Y	Y	Y	N	N	Y	Y	75
Doma et al. 2017 [29]	Y	Y	Y	Y	N	N	Y	Y	75
Doma et al. 2019 [30]	Y	Y	Y	Y	N	N	Y	Y	75
Drummond et al. 2005 [22]	Y	Y	Y	Y	N	N	Y	Y	75
Guimarães et al. 2020 [31]	Y	Y	Y	Y	N	N	Y	Y	75
Low et al. 2019 [19]	Y	Y	Y	Y	N	N	Y	Y	75
Marcello et al. 2017 [21]	Y	Y	Y	Y	N	N	Y	Y	75
Marcora et al. 2007 [18]	Y	Y	Y	Y	N	N	Y	Y	75
Palmer et al. 2001 [33]	Y	Y	Y	Y	N	N	Y	Y	75
Taipale et al. 2014 [23]	Y	Y	Y	Y	N	N	Y	Y	75
Taipale et al. 2015 [24]	Y	Y	Y	Y	N	N	Y	Y	75

Y—YES, N—No, U—Not clear. Q1: Were the inclusion criteria well defined? Q2: Have participants and context been described in detail? Q3: Were the measurements collected in a valid and reliable way? Q4: Were standardized and objective inclusion criteria used? Q5 Were any confounding variables found? Q6: Were strategies used to deal with confounding variables? Q7: Were the results measured validly and reliably? Q8: Was the statistical analysis used adequate?

### Study Characteristics

The experimental approach of 4 studies [19–22] presented as specific intervention protocols that aim to generate maximum "exercise-induced muscle damage" (EIMD), whereas 2 [23, 24] used characteristic protocols in order to generate post-activation potentiation (PAP). Other studies presented the intervention as a ST session, in which one used plyometrics combined with ST [25], five performed the intervention with combined training (combined strength exercises in the same session with aerobics) [26–30] and seven used a traditional ST session [31–37]. The summary of the characteristics of the studies included in the review is described in Table 2.

**Table 2:** Summary of the experimental design and results of the included studies

Study	*n*	Runner’s level	Training type	Protocols	Recovery Period	Variables		
						Neuromuscular	Physiological	Performance
Low et al. 2019 [23]	12	Trained	ST(PAP)	4 × 5RM band-squat jumps)	8 min	↑ DJ	↑ HR, ↔ RPE	↓ TCTT
Marcello et al. 2017 [25]	9	Trained	PRT	ST (3 × 5x85% barbell squats, Romanian deadlifts, barbell lunges; 5RM lateral lunge); PT (3 × 5 box jumps, depth jumps)	Immediately and 24 h	–	↑ VO_2_, ↓ RER	↑ RE
Blagrove et al. 2019 [4]	17	Trained	PT (PAP)	6 DJ	10 min	–	↔ HR, La, RPE ↑Readiness	↓ RE, ↔ TTE
Drummond et al. 2005 [26]	10	Not specified level	ST + AE	3 × 10 × 70%1RM of 7 exercises + 25 min at 70% VO_2max_	5 min	–	↑ VO_2_, HR, RPE	–
Marcora et al. 2007 [22]	24	Moderately trained	PT (EIMD)	100 DJ	48 h	↓ P_T_	↑ DOMS, ↑ CK, ↑ La, ↑RPE ↔ VE. VT, VO_2_, VCO_2_, HR ↑RER	↓ Speed
Doma et al. 2014 [32]	15	Recreational	ST	STAI: Incline leg press—6 × 6, Bench press—4 × 6, Bench pulls—4 × 6 STLI: Incline leg press—6 × 20, Bench press—4 × 20, Bench pulls—4 × 20	6 h	↓ P_T_	↔ VO_2_, HR, RPE ↔ RER	↓TTE
Taipale et al. 2014 [27]	12	Recreational	ST + AE	3 × 5x70-85%—Leg Press: 3 × 8- 10 × 30—40%; Squat: 3 × 5–8 × 70–85%; SJ: 3 × 8–10 × 30—40%	Immediately, 24 h and 48 h	↓ P_T_ CMJ	↑ CK, ↔ La	–
Doma et al. 2013 [29]	14	Recreational	ST + AE	6 × 6 Leg press, leg extension and leg curls 4 × 6; Running session: 70%-90% of VT_2_ and 110% of VT_2_	6 h	↓ P_T_	↑ RPE	↔ C_R_ ↓ TTE
De Souza et al. 2011 [36]	11	Recreational	ST	5 × 5RM leg press, 2 × 15RM leg press	Immediately	–	↔ VO_2_, HR, RPE	–
Taipale et al. 2015 [28]	12	Recreational	ST + AE	5–8/ 8–10 (70%-85% / 30%-40%) Maximal leg press—Explosive leg press- Squat—Squat Jump-Calf raise	Immediately	↓ P_T_	↔ VO_2_, HR, ↓ La	↑ RE ↓ Stride length ↔ distance, Speed
Palmer et al. 2001 [37]	9	Trained	ST	3 × 8RM—Bench press; Squat; Upright Row; Dead Lift; Seated Row	Immediately, 1 h, 8 h, 24 h	↓ P_T_	↔ VE, HR, La, RPE ↑La	↔ Stride length ↓ RE
Doma et al. 2015 [31]	14	Not specified level	ST	6 × 6RM squats, single-leg leg press, leg extension and leg curls	24 h and 48 h	↓ CMJ	↓ DOMS ↑ CK, VCO_2_, HR, RPE ↔ VO_2_	–
Doma et al. 2017 [34]	12	Not specified level	ST	3 × 6RM, squats on a Smith machine, horizontal leg press, leg extension and leg curls	Immediately, 24 h, 48 h	↑ CMJ	–	–
Burt et al. 2013 [20]	9	Recreational	ST (EIMD)	10 × 10x80% BW, Smith machine squats	24 h and 48 h	↓ P_T_, CMJ, DJ, SJ	↑ DOMS, VO_2_, La, VE, RPE ↔ HR	↓ Stride length
Burt et al. 2012 [19]	10	Not specified level	ST (EIMD)	10 × 10x80% BW Smith machine	24 h and 48 h	↓ P_T_	↑ CK, DOMS, VO_2_, RPE ↔ HR, La	↓ Stride length
Burt et al. 2014 [21]	8	Not specified level	ST (EIMD)	100 × 80% BW Smith Squat	24 h and 48 h	↓ P_T_	↑ CK, DOMS, VE, VO_2_, HR, La, RPE	–
Guimarães et al. 2020 [35]	18	Not specified level	ST	21 × 15	30 min, 24 h, 48 h, 96 h and 144 h	–	–	↑ TCTT
Doma et al. 2013 [30]	12	Trained	ST + AE	6 × 6RM leg press + 4 × 6RM leg curls and leg extension; Endurance: three incremental stages—70, 90 and 110% VT2	6 h and 24 h	↓ P_T_	↑RPE	↑ C_R_ ↓ TTE
Doma et al. 2020 [33]	10	Not specified level	ST	3 × 6RM squats on a Smith machine, horizontal leg press, leg extension and leg curls	Immediately, 24 h, 48 h	↓ CMJ	↑ DOMS, VE, VO_2_, VCO_2_, HR ↔ CK ↑ RER	–

*ST* Strength training, *PAP* post-activation potentiation, *DJ* drop jump, *RM* repetition maximum, *HR* heart rate, *RPE* rating of perceived exertion, *TCTT* time to complete the time trial, *PRT* plyometrics and resistance training, *PT* plyometric training, *P*_*T*_ Peak torque, *VO*_*2*_ Oxygen consumption, RER respiratory exchange ratio, *La* lactate, *RE* running economy, *TTE* time to exhaustion, *RE* running economy, *AE* aerobic exercise, *EIMD* exercise-induced muscle damage, *DOMS* delayed-onset muscle soreness, *VT* tidal volume, *VCO*_2_ carbon dioxide production, *VE* ventilation, *CMJ* countermovement jump height, *CK* creatine kinase, *SJ* squat jump, *BW* body mass, *C*_*R*_ cost of running, *VT*_2_ ventilatory threshold

**Fig. 3:**
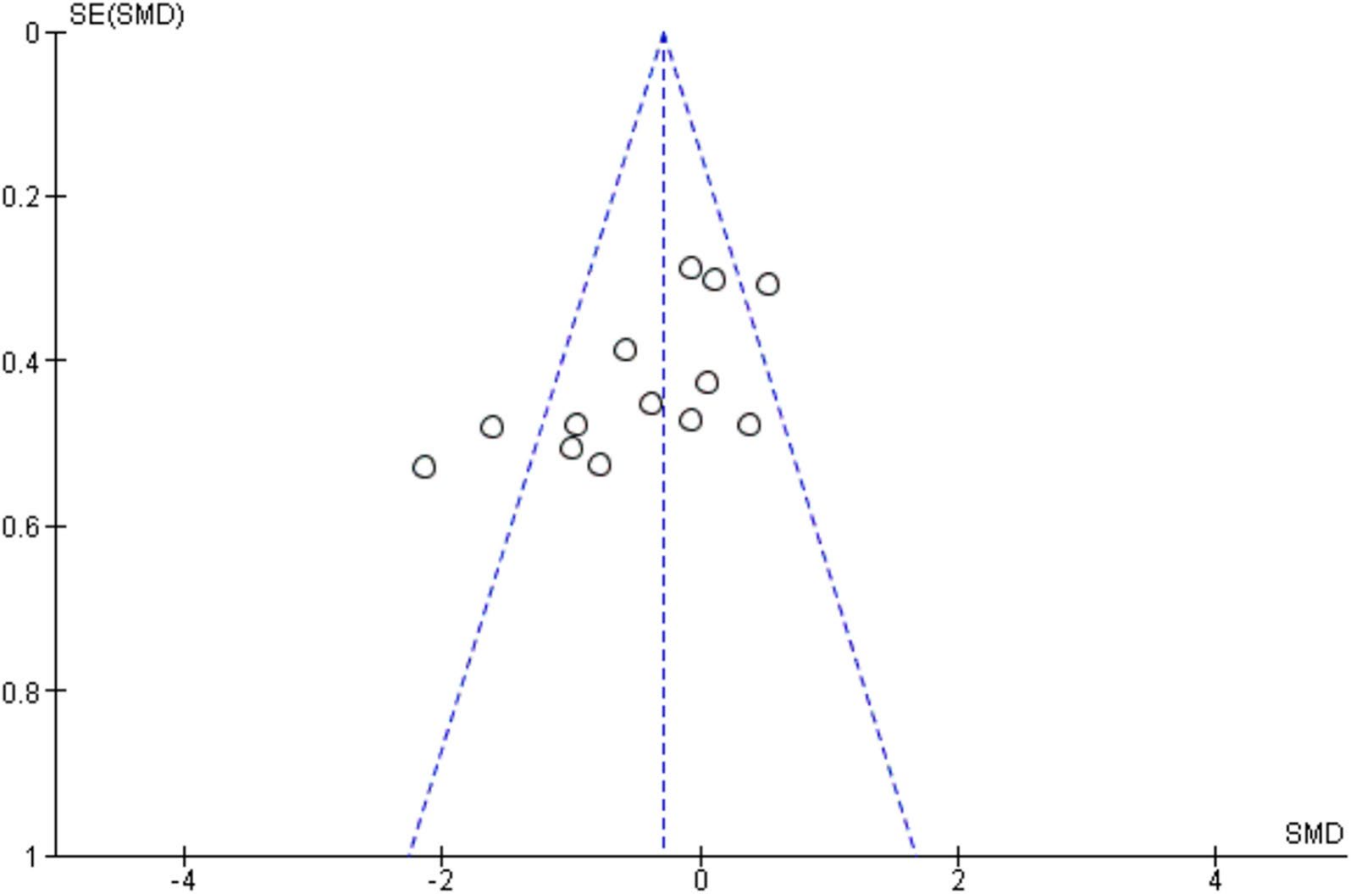
Funnel plot of risk of bias of studies included in the meta-analysis

Ten studies [4, 23, 25–28, 33–37] analyzed the outcome immediately after the intervention. Eleven [19–21, 25, 27, 29–31, 33–35, 37] also analyzed outcomes 24 h later, whereas 9 [19–22, 27, 31, 33–35] also included outcomes 48 h later. The funnel plot shows symmetrical results and high concentration at the top of the pyramid indicating low risk of bias of studies included in the meta-analysis (Fig. 3).

### Meta-Analysis

#### Neuromuscular and Physiological Variables

**Fig. 4:**
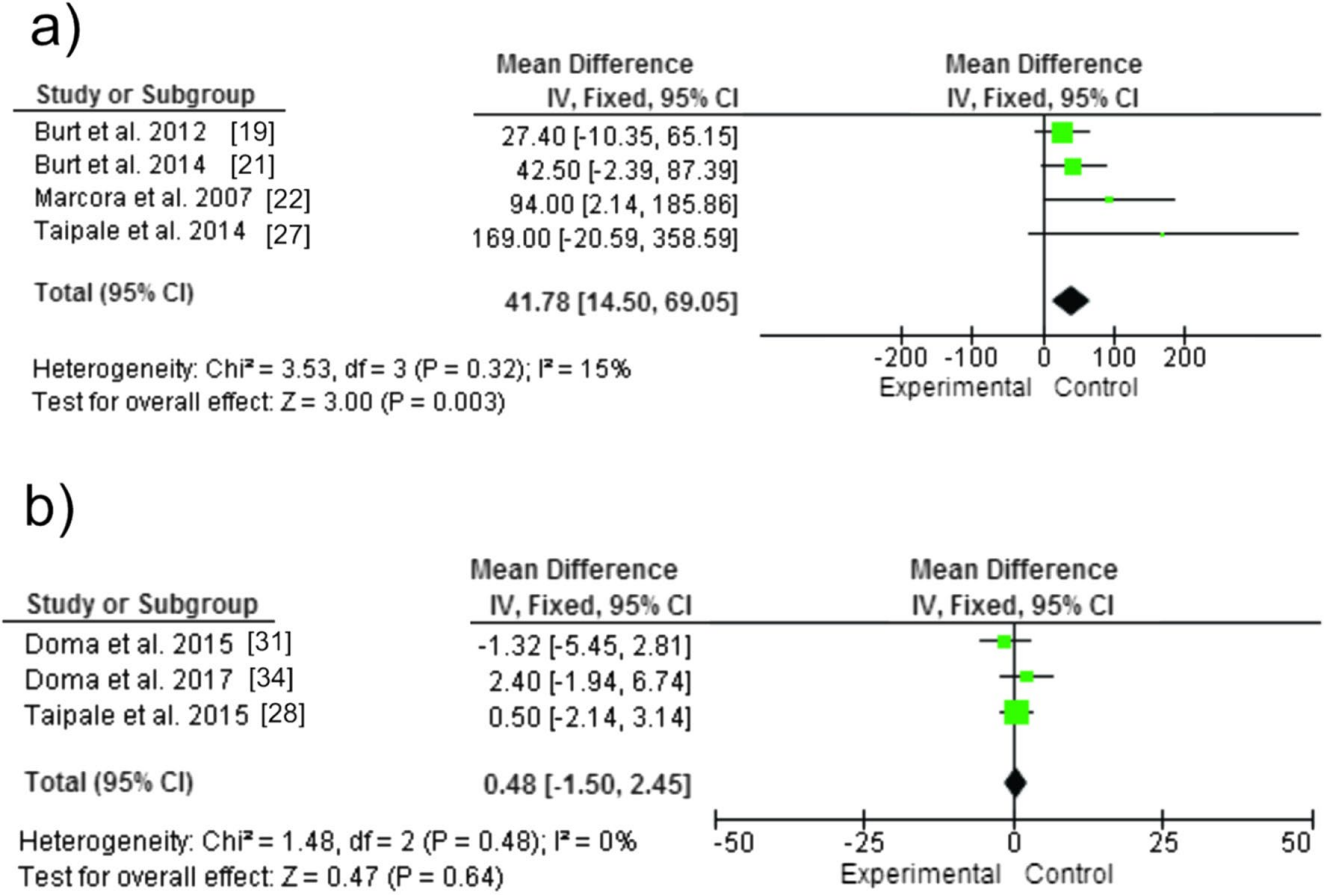
Acute effects of strength training session on neuromuscular variables in subsequent running session: **a** Peak torque (P_*T*_) **b** Countermovement jump (CMJ)

The meta-analysis showed that there was no significant difference between the experimental and control groups for vertical jump with countermovement (CMJ): SMD [95% CI] = 0.48 [− 1.5 to 2.45], Z = 0.47, *p* = 0.64). Whereas peak torque (P_*T*_) showed a significant difference between groups, with deleterious effects for the experimental group: SMD [95% CI] = 41.78 [14.50 to 69.05], Z = 3, *p* = 0.003). Drop jump (DJ) was evaluated in only one study, which was not included in the meta-analysis, of PAP characteristics [23] and showed a significant positive effect (*p* = 0.02). Figure 4 graphically presents the respective analyses.

**Fig. 5:**
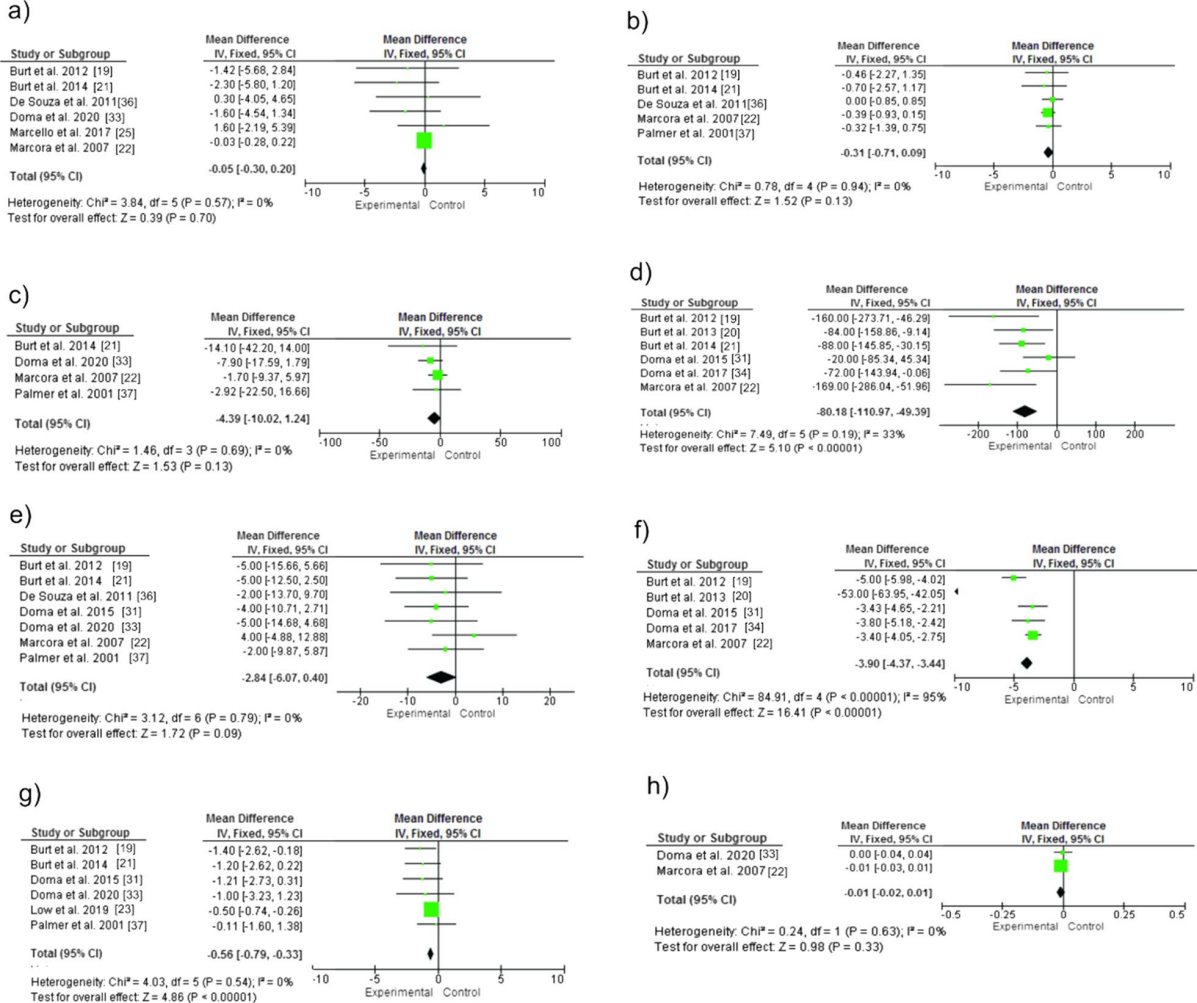
Acute effects of strength training session on physiological variables in subsequent running session: **a** Oxygen consumption (VO_2_) **b** Lactate (La) **c** Ventilation (VE) **d** Creatine kinase (CK) **e** Heart rate (HR) **f** Delayed muscle pain (DOMS) **g** Rating of perceived exertion (RPE) **h** Respiratory exchange (RER)

The meta-analysis showed (Fig. 5) a significant difference between groups for delayed-onset muscle soreness **(**DOMS) with greater effect on the experimental group: SMD [95% IC] =  − 3.90 [− 4.37 to − 3.44], *Z* = 16.41, *p* < 0.0001. For CK, there was a significant difference with greater effect on the experimental group: SMD [95% CI] =  − 80.18 [− 110.17 to − 49.39], *Z* = 5.1, *p* < 0.0001). For lactate (La) there was no significant difference between groups: SMD [95% CI] =  − 0.31 [− 0.71 to 0.09], *Z* = 1.52, *p* = 0.13. For the rating of perceived exertion (RPE) there was a significant difference between groups with greater effect on the experimental group: SMD [95% IC] =  − 0.56 [− 0.79 to − 0.33], *Z* = 4.86, *p* < 0.0001). As for heart rate (HR), there was no significant difference between groups: SMD [95% CI] =  − 2.84 [− 6.07 to 0.40], *Z* = 1.72, *p* = 0.09. Oxygen consumption (VO_2_) also showed no difference between groups: SMD [95% CI] =  − 0.05 [− 0.30 to 0.20], *Z* = 0.39, *p* = 0.70. Regarding respiratory exchange (RER), the analysis did not show significant differences between groups: SMD [95% CI] =  − 0.01 [− 0.02 to 0.01], *Z* = 0.98, *p* = 0.33. For minute ventilation (VE) no significant differences were observed: SMD [95% CI] =  − 4.38 [− 10.02 to 1.24], *Z* = 1.53, *p* = 0.13. A low evidence of heterogeneity (Ch^2^ and tau^2^) and inconsistency (I^2^) was found.

#### Performance

Performance data were not included in the meta-analysis because they did not objectively present mean values and standard deviations at the time of interest; were collected in only one study; or because they were not collected at similar intervals between intervention and outcome. The length of the stride was evaluated in three studies. In two of them [19, 28], there was a significant reduction in stride length (2.24 ± 0.26 to 2.2 ± 0.28 m and from 1.27 ± 0.07 to 1.22 ± 0.09 m, respectively), whereas the other [37] found no differences. The time to exhaustion (TTE) was analyzed in four studies. In three studies [29, 30, 32] (traditional ST protocols), there was a reduction in time for the experimental group, whereas in one study [24] there were no significant differences. As for running speed (km/h), two studies [22, 28] evaluated this variable and in only one study [22] there was a significant change for the experimental group (Pre 13.9 ± 1.7-Post 13.6 ± 1.7 km/h).

The time to complete one kilometer was evaluated in two studies. Low [23] used a PAP protocol in which a significant reduction in time was observed, whereas in Guimarães [35] there was an increase in the time to complete 5 km when comparing the values pre vs post 30 min and post 48 h (*p* = 0.02 and *p* = 0.04, respectively). As for the distance covered, Taipale [28] used combined training protocols and reported that there was no significant difference between control or experimental conditions (11.6 ± 0.9 / 11.7 ± 1.0 km).

## Discussion

The objective of this systematic review and meta-analysis was to synthesize the findings in the literature from 1995 to April 2021 regarding the acute effects of ST, in its different modalities (traditional, explosive, plyometrics and concurrent) on neuromuscular, physiological and performance responses in runners. It was verified that performing a ST session, even at high intensity (> 80%RM or 6 RM), with a 24-h interval for a submaximal running session does not change the vertical jump response, physiological respiratory capacity, performance training and submaximal running.

The literature on concurrent training, especially on the effect of aerobic training on ST adaptations, is extensive and well-developed in sports and clinical populations. [1, 38–40]. In addition, ST and its neuromuscular and mechanical adaptations already present consolidated knowledge about the benefits in the performance of aerobic modalities [5, 8]. On the other hand, knowledge of the immediate and short-term effects of a ST session on the indirect and directly related variables to running performance is necessary and should be part of the combined training intersection planning.

We hypothesized that session protocols that require higher training load (repetitions, sets, load, execution speed), higher density and proximity to concentric failure generate greater deleterious effects on the subsequent running session [10]. Thus, the hypothesis was partially rejected. The results showed that the variables P_T_, DOMS, CK, RPE had a deleterious effect for the experimental group that performed ST. Although it was expected that studies that performed a protocol to promote exercise-induced muscle damage would show this behavior [19–22], this fact has not been confirmed in plyometrics and traditional training protocols.

As for the outcome of the neuromuscular variables, it is known that the accumulated fatigue after the ST session is due to central factors (reduction in the levels of recruitment of motor units, as well as their activation frequencies) [41], local and peripheral alteration of the structure of the sarcolemma, accumulation of metabolites in the blood flow and ionic imbalance [42]. Consequently, it is expected that there will be changes in the mechanical aspects during the session. Among these variables, high levels of force, such as P_T_, measured at fixed angles in isometric action, decreased. As for the CMJ, a variable related to jumping ability, sensitive to fatigue and neuromuscular status [43, 44], with characteristics of dynamic contraction (fast eccentric and concentric phase) in contrast to P_T_, it did not present a significant difference. This observation can be justified according to studies that used combined training protocols and traditional intensities of ST (6RM) [31] or ST combined with explosive exercises (30% to 40% of 1RM) [28] in study design with 24 h interval. Suggesting a greater need for recovery following the initial session of lower segments [31]. As for those studies that analyzed P_T_, three used EIMD protocols, such as the 10 sets of 10 repetitions at 80% of 1RM in the Smith squat in the study of Burt [19]. This protocol showed higher values in the variables of late muscle pain, swelling and stiffness, caused by disruption of the intracellular structure, sarcolemma, extracellular matrix and impaired muscle function [45].

For lactate levels, no statistically significant difference was observed in the meta-analysis [19, 21, 22, 36, 37]. Changes in La metabolism after an ST sequence do not seem to show consistent results, especially when evaluated during submaximal aerobic exercise. In some studies, the values of this variable did not show statistically significant differences [22, 46], whereas in others, significant differences were observed [47]. De Souza [36] points out that although there was a significant change in La immediately after ST in the two strength protocols used (maximum strength and endurance strength), there was no change after the first km of running (control, maximum strength, and endurance strength (2.7 ± 0.8, 2.7 ± 1.2, and 3.2 ± 1.8 mmolIS·L^−1^, respectively) even when the run was immediately afterward. Increased blood La level is associated with high intensity exercise due to higher anaerobic metabolism [48, 49], in contrast, it is believed that in the studies evaluated, the runners had a greater predominance of the aerobic zone or resynthesis handsome lactate during submaximal running [50].Ventilatory variables such as VE, VO_2_, RER and running economy (RE kcal/min/kg or kJ/min/kg and C_R_) were not affected by the previous ST session. The effort of running at a speed corresponding to the oxygen consumption in submaximal efforts (around 55 to 75% of VO_2_max) seems not to be affected by the ST session, even in sessions that used EIMD protocols [19, 22]. Other studies that evaluated VO_2_ after ST in aerobic modalities such as cycling or cycle ergometer showed no change [47, 51–53] in accordance with the result verified in this meta-analysis. The results indicate that the performance of runners after sessions of EIMD and ST seem not to be mediated by cardiorespiratory and metabolic responses to exercise. Mechanical efficiency has been reported as an important determinant of running economy. However, 1 study [37] showed no change in stride length and 3 [20, 28, 39] showed a reduction. Thus, variations in running technique did not favor running economy, further suggesting that any disturbance in this efficiency will subsequently increase aerobic demand [37].

Regarding direct performance measures, few studies have evaluated this variable. Of the selected articles, although statistically different values were observed between the experimental and control groups for speed (km/h) during treadmill running at 1% incline in one study [22]; stride length [19, 28]; and in the time to finish a 5 km run, in absolute values this difference was relevant immediately after the session, but irrelevant after 48 h (20.63 ± 2.42 min before intervention; 22.40 ± 2.86 min after 30 min and 21.26 ± 2.56 min after 48 h) [35].

The exception seems to be in studies that analyzed responses to TTE followed by high-intensity strength sessions (4 × 6RM), in which there was a significant difference with deleterious effects for groups that performed ST concurrently with the running session within 24 h later of the ST session. Doma [30] performed a combined training on the same day: a ST session and, 6 h later, a running session, and applied the TTE the following day (24 h after the ST session and 18 h after the running session). In this case, the high intensity of workload in the ST and the short time between intervention and outcome assessment may have been the main reasons that caused this drop in performance. In the same study, the C_R_ of the running session was also higher, demonstrating from 6 h deleterious changes in running economy for the group that performed ST.

The present review has the following limitations. Due to the low quantity of studies we were unable to perform a robust analysis of the differences between the effects immediately, and at 6, 10, 24 or 48 h. In general, the main outcome observed was there was a reduction in P_T_ independent of the evaluation immediately, and at 6, 24 and 48 h [16, 19, 21, 22, 27, 28, 30, 32, 37]. Regarding the other variables, we can still notice a discrepancy between the results, which makes practical implications difficult. For example, in the study by Palmer et al. [37] after ST: 3 × 8RM—bench press; squat; upright row; dead lift; seated row, no changes were observed in VE, HR, La, RPE and stride length after 24 h. In contrast Burt et al. [19] after performing ST: 10 × 10x80% body mass Smith machine showed an increase in the evaluations of CK, DOMS, VO_2_, RPE and reduction of stride length after 24 and 48 h. The other variables presented heterogeneous conditions for this evaluation. Furthermore, with the increase in the number of publications on the subject we suggest further studies with a specific population of runners (i.e., recreational or athletes). Some studies proposed different aims and experimental protocols which lead to heterogeneous results. The strengths of this review were the variety of ST protocols added, the sample size, and the reasonable quality of the studies, showing that it is possible for athletic coaches, professionals, and researchers to expect results in line with those presented in this study. It is suggested that more research evaluate direct performance measures, a point considered limited in this study. In addition, studies that use protocols with lower loads, training with body weight and multicomponent strategies are also suggested.

### Practical Implications

In summary, although many runners use ST prior to participating in running competitions and during recreational practice, has not been shown to acutely improve performance. This complementary preparation strategy, commonly used to improve neuromuscular adaptation, showed a substantial deleterious effect. So, care to decrease potential muscle damage before competition is recommended. The acute use of ST in combined training programs (i.e., cross training exercises) could be indicated as aerobic benefit and little indicated for immediate performance gains for the runner. Thus, regular (chronic effect) and alternating day ST sessions remain recommended.

## Conclusion

Performing acute strength-training session in conjunction with endurance-training decrease the peak torque, increase delayed-onset muscle soreness, creatine kinase and rating of perceived exertion but not affect submaximal running sessions. In addition, performing these modes of training showed low impact in the countermovement jump, ventilation, oxygen consumption, lactate and heart rate.

## Data Availability

Data and materials support published claims and field standards.

## Abbreviations

ST: Strength training
P_T_: Peak torque
CK: Creatine kinase
DOMS: Delayed-onset muscle soreness
RPE: Rating of perceived exertion
CMJ: Countermovement jump
VE: Ventilation
VO_2_: Oxygen consumption
La: Lactate
HR: Heart rate
RM: Maximum repetition
EIMD: Exercise-induced muscle damage
PAP: Post-activation potentiation
JBI: Joanna Briggs Institute
SMD: Standardized mean difference
CI: Confidence intervals
Ch^2^: Cochran Test
tau^2^: Tau-square
I^2^: Inconsistency
